# Unveiling Visual Acuity in 58,712 Four-Year-Olds: Standardized Assessment Defined Normative Visual Acuity Threshold

**DOI:** 10.3390/vision8020039

**Published:** 2024-06-19

**Authors:** Mirjana Bjeloš, Mladen Bušić, Benedict Rak, Ana Ćurić, Biljana Kuzmanović Elabjer

**Affiliations:** 1 University Eye Department, Reference Center of the Ministry of Health of the Republic of Croatia for Pediatric Ophthalmology and Strabismus, Reference Center of the Ministry of Health of the Republic of Croatia for Inherited Retinal Dystrophies, University Hospital “Sveti Duh”, 10000 Zagreb, Croatia; dr.mbjelos@gmail.com (M.B.); benedict.rak@gmail.com (B.R.); akrizanovic25@gmail.com (A.Ć.); belabjer@kbsd.hr (B.K.E.); 2 Faculty of Medicine, Josip Juraj Strossmayer University of Osijek, 31000 Osijek, Croatia; 3 Faculty of Dental Medicine and Health Osijek, Josip Juraj Strossmayer University of Osijek, 31000 Osijek, Croatia

**Keywords:** amblyopia, registries, child, screening, visual acuity

## Abstract

The purpose was to define the threshold of normal visual acuity (VA), mean monocular and binocular VA, and interocular difference in the uniform cohort of healthy four-year-old children. All the children were recruited from the Croatian National Registry of Early Amblyopia Detection database. LEA Symbols^®^ inline optotypes were used for VA testing at near and distance, binocularly and monocularly. The pass cut-off level was set to ≤0.1 logMAR. The final sample consisted of 58,712 four-year-old children. In total, 83.78% of the children had unremarkable results, and 16.22% of the children were referred to examination. Of those, 92% of the children were referred due to binocular, and 8% of the children due to monocular causes. The children referred due to binocular causes demonstrated a VA of 0.3 ± 0.24, while the children referred due to monocular causes 0.6 ± 0.21. The ROC curve analysis defined the uniform cut-off value for a normative VA of 0.78. We analyzed the largest uniform cohort of 58,712 children, and have determined normative data for binocular and monocular VA tested with gold standard logMAR chart in four-year-old children. The results presented here established no reasoning to further utilize historical protocols in testing VA in preschool children aged ≥ 4 years.

## 1. Introduction

Vision disorders are acknowledged to be the leading cause of handicapping conditions in childhood [1,2]. The key visual function and one of the most important indicators of normal visual development is visual acuity (VA) [1,2].

Amblyopia, the most prevalent vision disorder in preschool children, is a neurodevelopmental disorder of the visual cortex that results from retinal image degradation during the maturation of the visual system. The loss of vision in amblyopia is most frequently presented as VA decline. Although the majority of publications acknowledge amblyopia as a monocular disorder predominantly [3,4], the exact ratio of monocular versus bilateral amblyopia is still not determined [5].

Amblyopia has considerably different prevalence related to geographic areas, ethnicity, and age [6]. Screening methodologies should directly target amblyopia and not the risk factors, thus the precise quantification of vision is of utmost importance in defining amblyopia whereas the gold standard for amblyopia screening is VA testing [7].

However, a large variety of screening methodologies and inconsistent protocols for referral, including different types of optotypes, crowding format (linear charts, crowding bars, surrounding contours, and surrounding pseudo-optotypes), and optotype size progression, intensifies the debate on determining the most effective protocol for vision testing [8,9,10,11]. The unique definition of amblyopia accepted for research has not reached a consensus, further challenging the standardization of the screening protocols. Due to developmental changes in crowding in central vision in childhood, only inline testing charts should detect the adverse effects of abnormal visual input in childhood [12].

Between September 2011 and June 2014, the Zagreb Amblyopia Preschool Screening (ZAPS) prospective study tested near and distance VA, binocularly and monocularly, using Lea Symbols inline [12]. Of 15,648 children aged 48–54 months attending kindergartens in the City of Zagreb, 78.04% of the children passed the screening test with the cut-off value VA ≤0.1 logMAR [12]. The results evidenced that the estimated prevalence of amblyopia was 8.1% [12], considerably higher than reported elsewhere [6]. The age-specific normative threshold was accordingly defined at ≤0.1 logMAR for four-year-old children using Lea Symbols inline [12].

Based on ZAPS results as of June 2015, the vision screening of all four-year-old children performed in ophthalmologists’ practices was introduced as a national health policy, followed by the implementation of the Croatian Registry of Early Amblyopia Detection (CRO–READ) on 1 June 2017. This user-friendly online application is accessed using the secured username and password of the ophthalmologists who perform screening and treatment [13]. Ophthalmologists performing the screening are obliged to enter the results, as only the records evidenced with the Registry will be financially reimbursed.

The primary aim of this study was to define the threshold of normal VA, mean monocular VA, mean binocular VA, and interocular VA difference in the cohort of healthy four-year-old children, the participants of the Croatian National Preventive Program of Early Amblyopia Detection (CRO-PPEAD) through the analysis of data from the Registry for the period 2017–2022.

## 2. Materials and Methods

All the study procedures adhered to the institutional and governmental legislations regarding ethical principles for medical research involving human subjects, and the tenets of the Declaration of Helsinki. Ethics approval for this study was obtained from the Ethics Committee of the University Hospital “Sveti Duh”, Zagreb, Croatia (registry number 03-4104).

### 2.1. Sample Size

#### 2.1.1. CRO–READ

All the children were recruited from the CRO–READ electronic database for the period 2017–2022 [13]. Reporting to the Registry is internet-based, using the homepage https://ambliopija.hzjz.hr/index.php/hr/ located at the Croatian Institute for Public Health. Four data sheets were provided: the entry form, screening examination form, comprehensive examination form, and follow-up examination form. The sheets were designed in the multiple-choice format, limiting the option of entering free text. The screening sheet aggregated data on binocular and monocular near and distance VA, optotypes used for testing, and advocated for further evaluation if needed (Figure S1).

The comprehensive examination page contained information about whether the child was diagnosed with amblyopia, and data about further evaluation if needed. The screener has the possibility to enter other important remarks (Figure S2).

Data inputs were performed on the date of screening or comprehensive examination accordingly.

#### 2.1.2. Visual Acuity Testing

LEA Symbols^®^ Pediatric Eye Near and Distance Charts (Good-Lite, Elgin, IL, USA), presenting optotypes inline using the logMAR progression format to detect crowding, were used for VA testing at near (40 cm) and distance (3 m) [12]. Binocular testing was performed first. Monocular VA testing was conducted with the right eye approached first, with the non-testing eye occluded with an adhesive patch, following the protocol previously reported [12]. The examination was performed at near (40 cm), followed by distance (3 m) VA measurement. Beginning with the 30 M line, the examiner continued through descending lines requesting the child to read a single optotype per line until they replied falsely. When incorrect, the examiner presented optotypes two lines above the line failed and asked the child to read the whole line. Four out of five optotypes in a line accurately interpreted constituted the pass criterion for the line. The VA testing was performed by ophthalmologists under photopic conditions in the ophthalmologists’ practices. The pass cut-off level for four-year-old children was ≤0.1 logMAR. It is important to emphasize that no additional test beyond VA should be incorporated as part of the standardized CRO-PPEAD protocol.

To ensure the standardization of testing, the Ministry of Health adopted a validated protocol for visual acuity (VA) testing in 2016 [12], establishing it as the national standard for the CRO-PPEAD [13]. According to the mandates outlined in the document Croatian National Preventive Program of Early Amblyopia Detection [13], exclusive authorization for performing VA testing is granted to ophthalmologists. Prior to their enrollment in the CRO-PPEAD, ophthalmologists are required to attain a comprehensive understanding of the VA testing protocol and data administration procedures via the CRO-READ. Subsequently, they are obliged to procure formal authorization from the Ministry of Health to assume the role of screeners. The protocol is accessible at all times via a publicly available internet link [13]. Furthermore, to ensure uniformity, all ophthalmological offices and requisite equipment in Croatia are standardized in accordance with the Ordinance on the Minimum Requirements Regarding Premises, Workers, and Medical–Technical Equipment for Performing Health Activities, enacted by the Minister of Health [14].

#### 2.1.3. Definition of Unremarkable Result, Amblyopia, Incorrectly Coded Data, Referral Criteria, and Testability

A result was considered unremarkable if the child had binocular and monocular VA ≤0.1 logMAR tested at near and distance and otherwise unremarkable ocular status. If the binocular and/or VA in a single eye was 0.2 logMAR either at distance or near, this could be declared unremarkable at the examiner’s discretion.

The diagnosis of amblyopia was made only by performing a complete ophthalmological examination. The criterion for unilateral amblyopia held to ≥2 lines interocular difference after re-testing the child on the comprehensive eye examination wearing full cycloplegic correction, with the best corrected VA of >0.1 logMAR in the worse eye presented with amblyogenic factor. The amblyogenic factors were defined as follows: hyperopia ≥2.00 D spherical equivalent (SE); myopia ≥3.00 D SE; astigmatism at any axis ≥1.00 D; anisometropia ≥1.00 D difference in hypermetropia, ≥3.00 D difference in myopia, or ≥1.00 D difference in astigmatism in any meridian; antimetropia with ≥1.00 D SE in the hyperopic eye; strabismus at near and/or distance fixation or history of strabismus surgery; or history or present evidence of the visual axis obstruction. Bilateral amblyopia held to >0.1 logMAR in both eyes after re-testing the child on the comprehensive eye examination wearing full cycloplegic correction in the presence of a bilateral amblyogenic factor. The amblyogenic factors were defined as bilateral: high hypermetropia ≥4.00 D, myopia ≥6.00 D, astigmatism ≥2.00 D, or history or present evidence of the visual axis obstruction.

VA recorded > 0.2 logMAR in either test and labeled as an unremarkable result was considered an incorrectly coded result and was excluded from further analysis. The criterion for referral to complete eye examination due to bilateral cause held to a VA of >0.1 logMAR in both eyes, at near or/and distance testing.

The criterion for referral to complete eye examination due to monocular cause held to a VA of >0.1 logMAR in only one eye at near or/and distance testing.

If the child did not respond at all, the child was labeled as non-testable (NT) and proceeded as failed the screening. The NT children were excluded from the testability analysis. The children who gave answers inconclusively or for whom the VA measurement could not be completed but had at least one VA test recorded were addressed as partially testable (PT) and proceeded as failed the screening.

In case VA was as poor and it could not be determined, it was labeled as visual impairment (VI). Testability was defined for the PT children. The VA of monocular and binocular testing was analyzed, as well as the mean VA in the children referred due to bilateral and monocular causes. In the children referred due to bilateral causes, all the VA results was evaluated. In the children referred for monocular causes, the near and distance VA of the ametropic eye only were analyzed. Only four-year-old children were included in the final analysis.

#### 2.1.4. Exclusion Criteria

The participants with incorrectly coded data, VI, and NT were excluded from the VA analysis.

The criteria for referral to a comprehensive eye examination, irrespective of VA pass results, included suspected strabismus and ocular disease at the examiner’s discretion. Instances of referral at the examiner’s discretion were specifically documented in the CRO-READ under the section “other”, where screeners could also record their observations about the patients. These patients were excluded from further analysis.

### 2.2. Outcome Measures

The primary outcomes were to define the threshold of normal, mean monocular, and binocular VA in the four-year-old children who do not have ocular abnormalities that would be expected to reduce VA, mean VA in children referred due to bilateral and monocular causes, and the interocular difference of monocular VA grouped by sex.

The secondary outcomes were to define the estimated prevalence of amblyopia in the City of Zagreb, and the testability of the used protocol [12].

### 2.3. Data Analysis

The recorded VA was converted from logMAR to decimal notation using the VA conversion chart provided on the test chart due to easier mathematical evaluation. Data were analyzed using a large program developed in Visual Basic for the sole purpose of analyzing the information from the Registry. Of note is the main equation used to calculate the predicted prevalence of amblyopia.

The data for this analysis came from the noted VAs, which themselves in nature are ordinal. However, VAs were almost always noted quantized, mostly in steps of 0.1 (or sometimes smaller). For this reason, there were 39 inter-group ties involving 352,253 records, limiting rank-based analysis to a degree.

The general distribution of VAs in this dataset had a mean of 0.83 and median of 0.8, with 95% CI between 0.84 and 0.85. The skewness parameter was −1.75, and kurtosis was 2.35. The Kolmogorov–Smirnov test gave a statistic value of 0.304. These parameters, together with the visual inspection of histograms, showed that this data did not follow normal distribution. Due to the very substantial sample size, in this particular case, it should be possible to draw valid conclusions using parametric tests, as per the central limit theorem. Even so, the best approach was to use nonparametric tests for the nonparametric data analysis of medians instead of means, and tests like Kolmogorov–Smirnov and Mann–Whitney. The nonparametric tests were shown, but independent samples mean *t*-tests were performed as well, supporting the same conclusions as the nonparametric tests.

Kruskal–Wallis 1-way ANOVA test for independent samples was used to determine the possible differences between the left and right eyes, near and far vision; Kolmogorov–Smirnov test, Mann–Whitney U test, and Mood’s median test (modified chi-squared) were used to determine possible differences between sexes.

The diagnosis of amblyopia was made only by performing a complete ophthalmological examination.

Due to a main limiting factor, the fact that not all of the children who were screened and instructed to take the full examination actually followed through, or a record of it does not exist in the registry, it was not possible to directly calculate the prevalence or incidence of amblyopia correctly. Ideally, if all the patients that were sent to complete examination did so, and were recorded, then the formula for the true result would be the number of confirmed amblyopia cases divided by the total number of screened children, which would yield a wrong (too small) number, because not every child that was instructed actually did the complete examination. It could also be calculated as a fraction of amblyopic cases over the total number of children that had the complete examination, but this would yield a wrong (too large) number, as that would be the prevalence of amblyopia in a population of cases that failed the screening test, not the whole population. Instead, it was estimated using the fraction of amblyopes over the total number of screened children, multiplied by a correction factor that was the fraction of all the children that failed the screening test over the number of children that actually did the complete examination.

The prevalence of amblyopia (in percentage) was therefore calculated as follows:

[(number of children that failed the screening test) × (number of confirmed diagnosed amblyopes on complete eye examination) × 100]/[(number of children examined on complete eye examination × number of children examined on screening test)].

## 3. Results

### 3.1. Sample Size

For the period 2017–2022, there were 76,071 records identified in the CRO–READ, and 6186 were incorrectly coded. Out of the 69,885 eligible participants, 292 children were labeled with VI, 2574 NT, and 1385 PT. The age was not recorded in 2193 children. The final sample consisted of 58,712 four-year-old children (352,272 VA tests) (Figure S3). There was equal distribution of males and females: 50.9% and 49.1%.

Lea inline charts were used in more than 95.7% of the cases (Figure S4).

**Figure 1:**
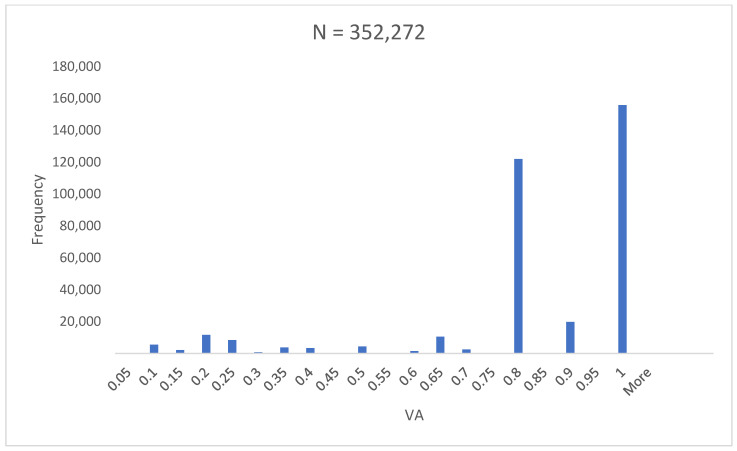
Histogram showing frequency of visual acuity results. Mean visual acuity equals 0.82 ± 0.24. VA, visual acuity; N, number of children.

The overall mean VA of 58,712 children was 0.82 ± 0.24 (Figure 1).

The proportion of children with VA ≤ 0.3 was 9.37%. A total of 49,191 children (83.78%) had unremarkable VA results, while 9521 (16.22%) children were referred to complete examination. Of those, 92% of the children were referred due to binocular causes, and 8% of the children due to monocular causes of VA decline (Figure S5).

Due to the fact that the total number of complete ophthalmological examinations of four-year-olds in Croatia was 352, it was impossible to draw valid conclusions on the prevalence of amblyopia.

### 3.2. Primary Outcomes

**Figure 2:**
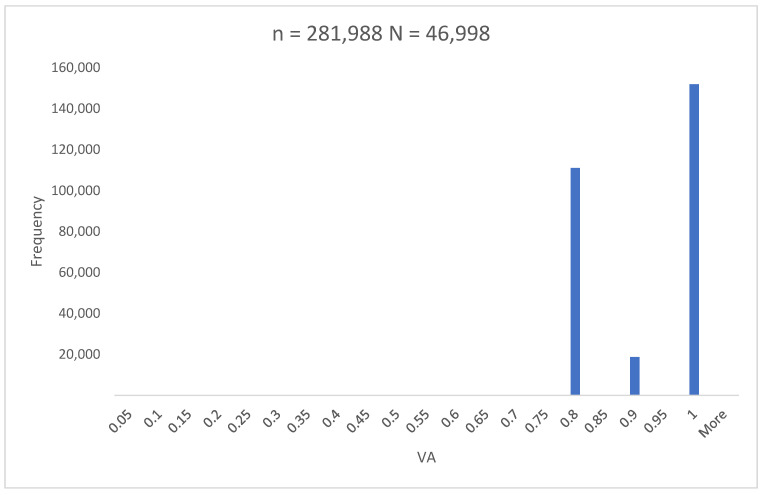
Histogram showing frequency of certain visual acuity. Mean visual acuity of children who had unremarkable screening result equals 0.91 ± 0.1 VA, visual acuity; N, number of children; n, number of VA exams.

The mean VA of the children who passed the screening was 0.91 ± 0.1 (Figure 2).

**Figure 3:**
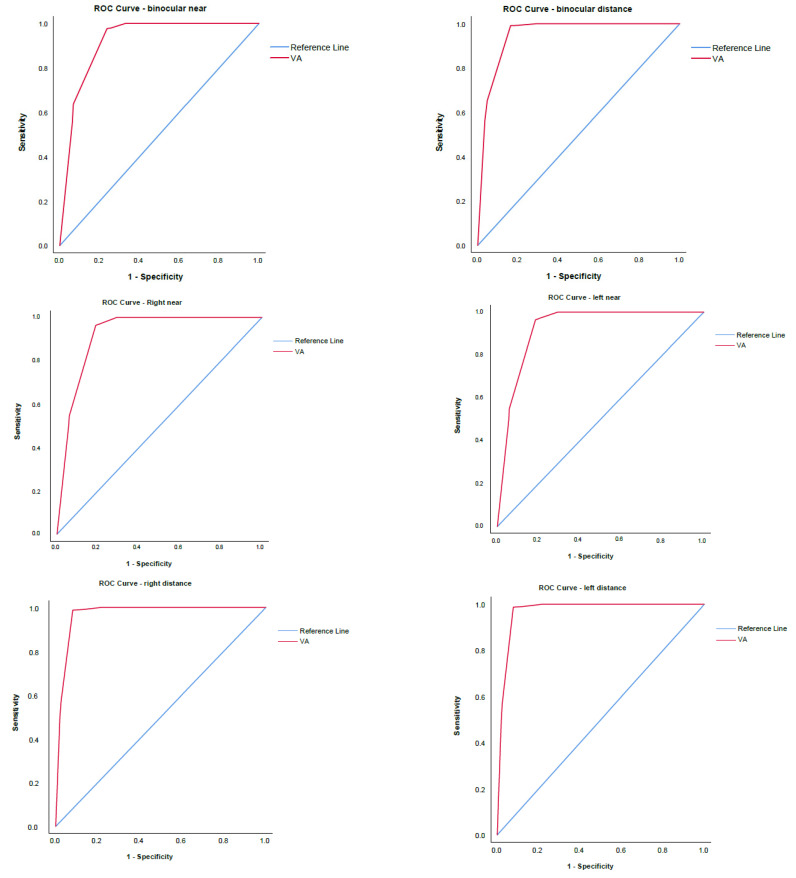
Receiver operating characteristic (ROC) curve for the visual acuity (VA) testing. For all the VA tests, the defined cut-off value was 0.78. The mean visual acuity and area under the curve are presented in Table 1. The 95% CI and Youden index are presented in Table 2. The methodology employed for determining the Youden index for each of the parameters is presented in Tables S1–S6. A very large area under the curve numbers was indicative of the high specificity and sensitivity of the tests.

A ROC analysis was performed on the six modalities of the VA tested. The goal variable was defined as unremarkable findings on the screening exam. The ROC curves defined the cut-off value for the normative VA result of 0.78 (Figure 3).

**Figure 4:**
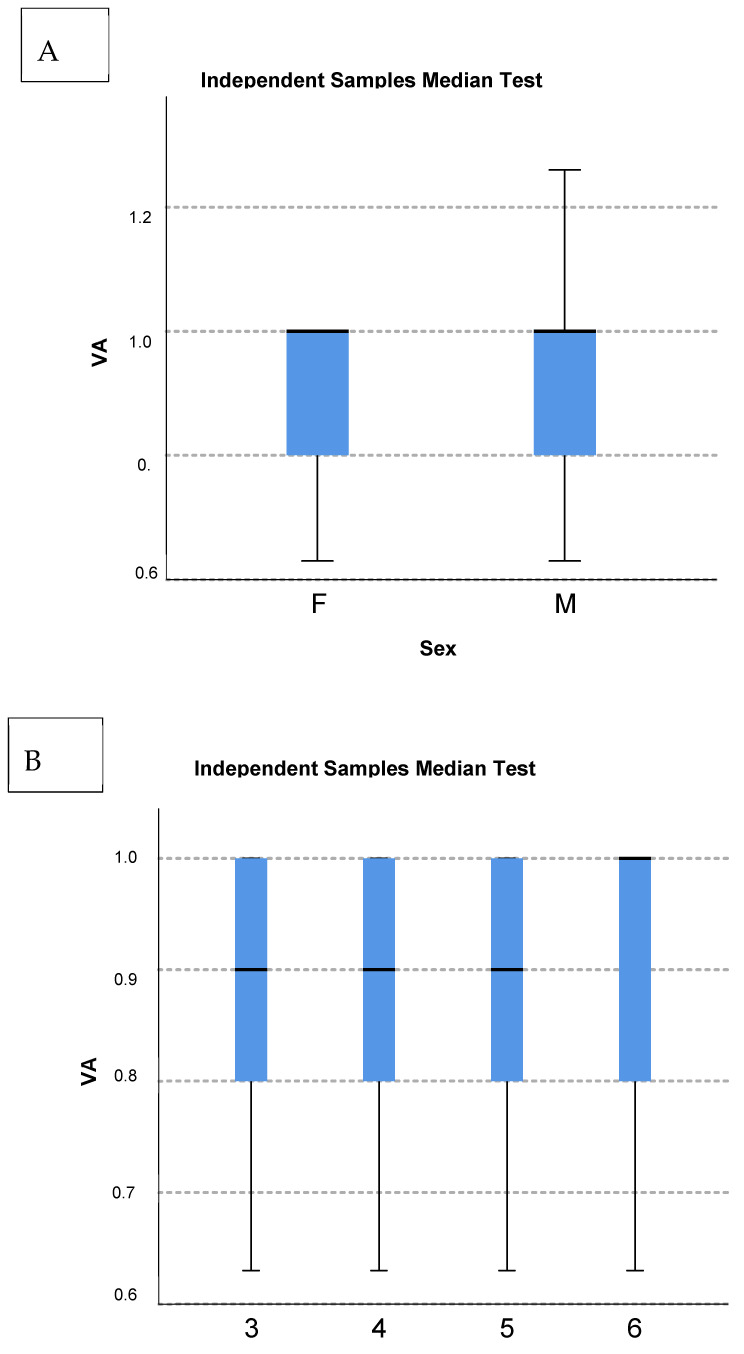

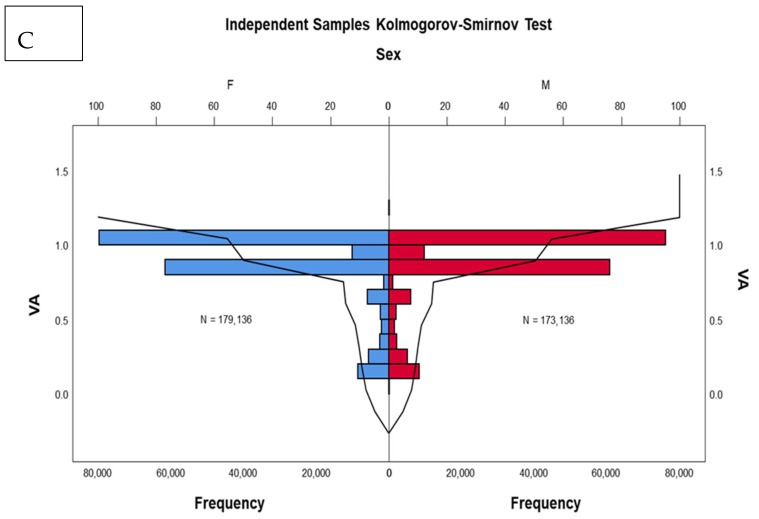
Visual acuity analysis and sex-based variations. (**A**) Sex-based variations in visual acuity. (**B**) Interocular visual acuity difference. (**C**) Sex-based variations on median and Kolomogorov–Smirnov test. VA, visual acuity; F, female; M, male; 3, right near; 4, right distance; 5, left near; 6, left distance; N, number of VA exams.

There was no interocular VA difference (*p* = 0.21) (Figure 4B). No sex-based variations were observed on the median and Kolmogorov–Smirnov tests (*p* = 0.99) (Figure 4A,C). Due to the large sample size, the Mann–Whitney test showed a 0.4% statistically significant rank value difference between sexes: a mean rank 173,136 for males and 175,802 for females. However, such a small figure has no real implication.

**Figure 5:**
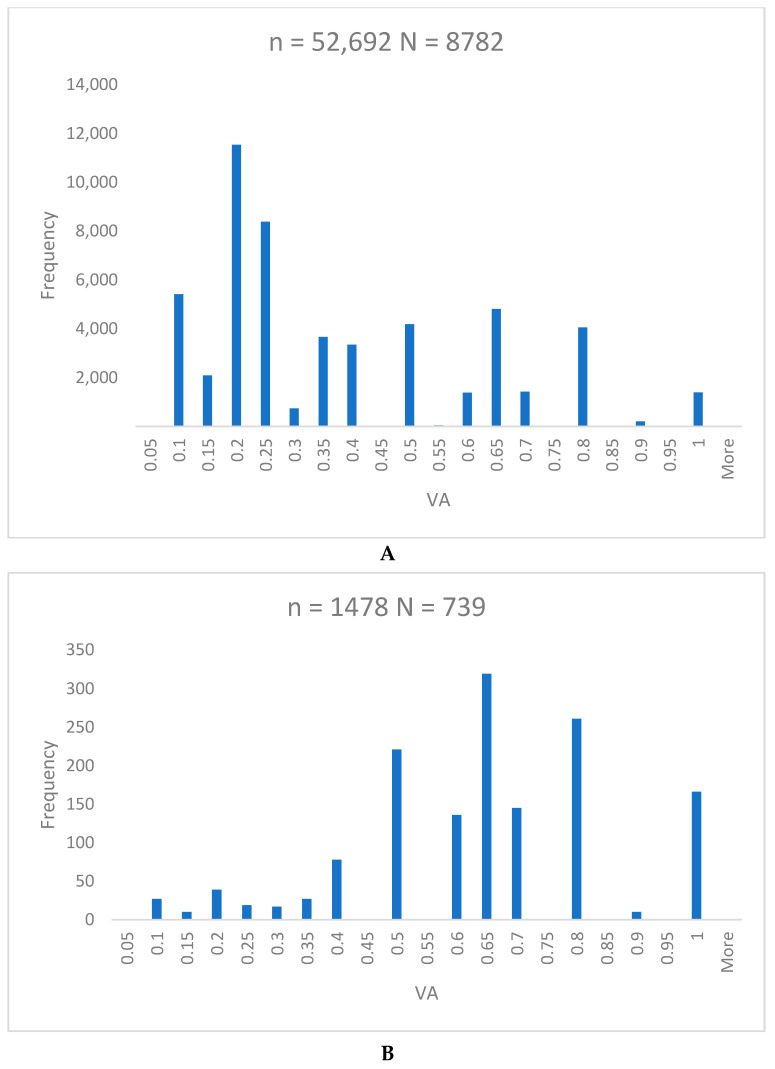
Histograms showing frequency of certain visual acuity (VA). Children referred due to bilateral causes (**A**), children referred due to monocular causes (**B**). VA, visual acuity; N, number of children; n, number of VA exams.

A total of 9521 children were referred to comprehensive examination. A total of 8782 children who were referred due to bilateral causes demonstrated an overall VA of 0.43 ± 0.27 (Figure 5A). The mean VA of the 739 children referred due to monocular causes was 0.64 ± 0.21 (Figure 5B). No statistically significant difference between the right and left eye was found (*p* = 0.28). The near and distance VA of the ametropic eye only were analyzed.

**Figure 6:**
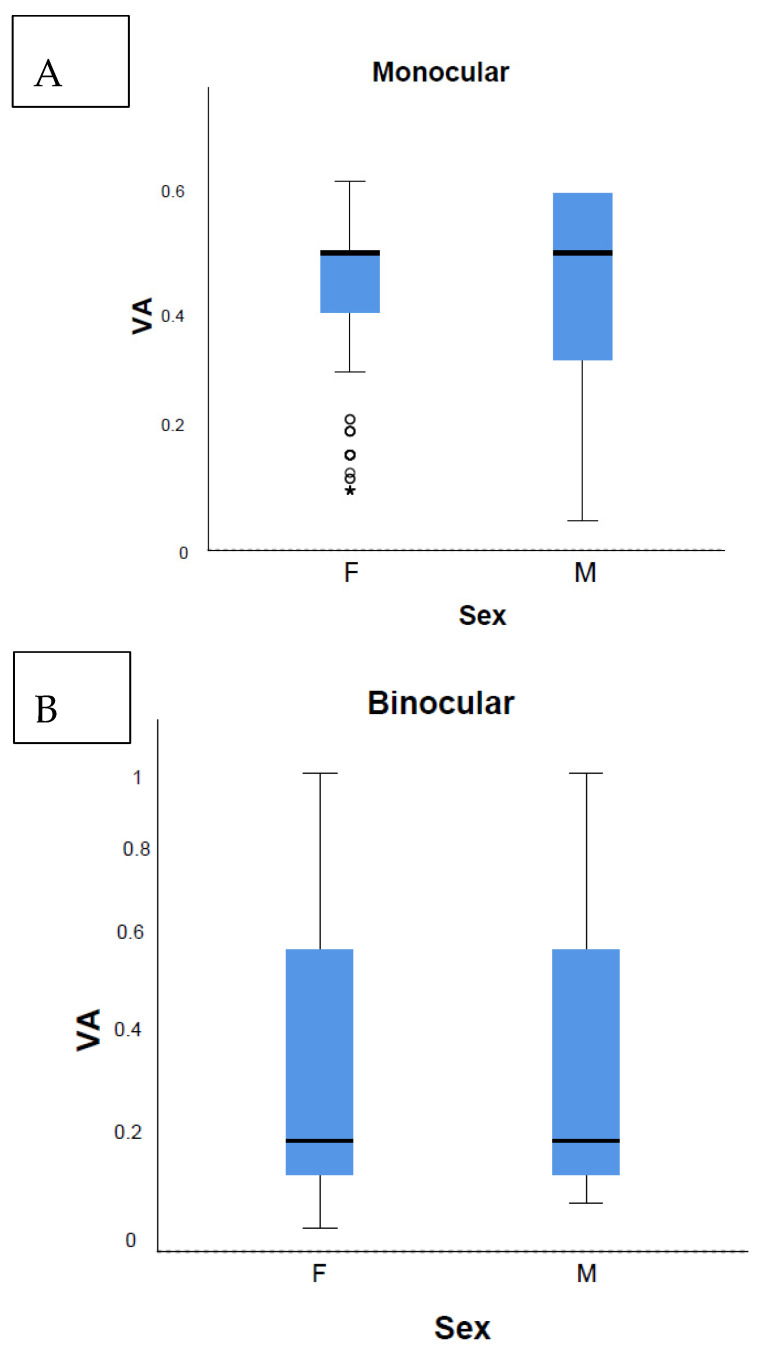

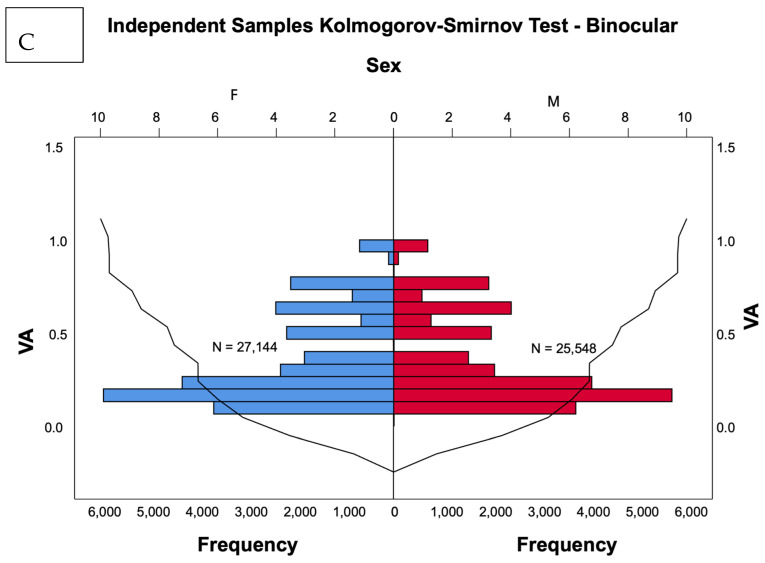
No sex-based variations were observed related to the distribution of monocular (**A**), and binocular causes (**B**), and in the total number of VA exams (**C**). VA, visual acuity; F, female; M, male, N, number of VA exams.

No sex-based variations were observed related to the distribution of monocular (*p* = 0.17) and binocular causes (*p* = 0.05) (Figure 6).

VA ≤ 0.3 was observed in 53.4% of the children and 7.6% of the children referred due to bilateral and monocular causes, respectively (Figure S6).

### 3.3. Secondary Outcomes

#### Non-Testable Children

Among the eligible children (N = 76,071), 292 were VI (0.38%), 2574 were NT (3.38%), while 1385 (1.82%) were PT. Of the 1385 PT and 2574 NT, 1159 (83.68%) and 2322 (90.21%), respectively, were four years of age. The distribution regarding gender revealed that males were significantly more NT and PT (*p* < 0.001) (Table 3).

VA in the group of PT children (N = 1159) is presented in Table 4.

## 4. Discussion

To the best of our knowledge, we analyzed VA in the largest uniform cohort of 58,712 healthy children and have determined the normative data for binocular and monocular VA tested with gold standard inline logMAR chart in four-year-old children. The validity of the results is strengthened by the use of a highly standardized protocol [12], and a uniform cohort of healthy children.

### 4.1. Age-Specific Normative Visual Acuity

The ROC curve analysis of binocular and monocular VA defined the uniform cut-off value for the normative VA of 0.78 justifying the criterion for referral to be set to VA > 0.1 logMAR (Figure 3, Table 1, Table 2 and Table S1–S6). The overall mean VA of four-year-old children who passed the screening of 0.91 ± 0.1 (Figure 2) suggests a quiescent period of VA development to the adult level onward. This finding corroborates the morphological studies of postnatal human retinal development that proved adult human cone density and foveal pit maturation were completed between four to six years of age [15].

Eye dominance was not observed (*p* = 0.21) (Figure 4). The theoretical significance of this result could be of importance to future research on sensory ocular dominance development.

It is proved that optotypes inline assembled on the logMAR principle are the most appropriate for amblyopia detection [12,16,17,18,19]. However, screeners still use different screening methodologies (Figure S4). Isolated single optotypes should not be used for screening as they overestimate VA [10,20].

Clinical trials, including emerging trials on digital therapeutic for the treatment of amblyopia, set the primary outcome of the increase in VA according to the Amblyopia Treatment Study (ATS) protocol [21,22,23,24,25]. The ATS protocol tests single bars flanked letter acuity acknowledged to overestimate VA [23,24], and distorts the criterion for defining monocular amblyopia set to interocular difference of ≥3 logMAR lines, potentially leading to implausible results [23]. The results presented here established no reasoning to further utilize historical protocols in testing VA in preschool children aged ≥ 4 years.

Given the current VA thresholds of 20/50, 20/40, 20/36, and 20/32, for four- and five-year-old children [21,23,26,27], this study confirmed that the chart- and age-specific threshold determining abnormal monocular VA in four-year-old children is >0.1 logMAR.

The Multi-Ethnic Pediatric Eye Disease Study group proposed a threshold of 20/40 for defining abnormal monocular VA using HOTV optotypes in children aged 48–59 months [21]. The proportion of children achieving the threshold was 99% [21]. However, this study revealed that 83.7% of the four-year-old children reached a VA of ≤0.1 logMAR. Hence, compared to our study results and earlier reports of ZAPS study [12], we proved the threshold 20/40 to be too low for the four-year-old age group as this criterion referred only 1% of the children to the complete ophthalmological examination, under-representing the number of false negatives. In 2014, the Vision in Preschoolers (VIP) study group concluded that the threshold levels associated with an increased risk of amblyopia recommended by professional organizations might be exceedingly lenient [28]. The American Academy of Ophthalmology guidelines define bilateral amblyopia as the best-corrected VA worse than 20/50 in both eyes for the age 3 to <4 years, worse than 20/40 in both eyes for the age 4 to <5 years, and worse than 20/30 in both eyes for the age ≥5 [23]. The Shenzhen Kindergarten Eye Study suggested VA cut-off criteria for further ophthalmic evaluation as 20/50 by the age of four, and 20/40 by the ages of five and six [26]. In support of the latter, the ROC curves here presented a defined cut-off value of 0.78 decimal, which is consistent with the 0.1 logMAR cut-off value (Figure 3, Table 1, Table 2 and Table S1–S6). No sex-related differences in VA were defined (Figure 6).

### 4.2. Development of Vision and Amblyopia Prevalence

We observed the intriguing phenomena among referred children. First, significantly less children were referred due to monocular causes (Figure S5). The second phenomenon emerged in the children who were referred due to binocular causes as they demonstrated VA of only 0.3 ± 0.24, contrary to the VA achieved in the children referred due to monocular causes of 0.6 ± 0.21 (Figure 5). No statistically significant difference between the right and left eye was found (*p* = 0.21) (Figure 5). No sex-based variations were observed related to the distribution of monocular (*p* = 0.17) and binocular causes (*p* = 0.05) (Figure 6).

VA ≤ 0.3 was observed in 53.4% of the children and 7.6% of the children who were referred due to binocular and monocular causes, respectively (Figure S6). Although the majority of publications acknowledge amblyopia as a unilateral disorder predominantly [3,4], the exact ratio of unilateral versus bilateral amblyopia is still not determined [5]. The Baltimore Pediatric Eye Disease Study revealed decreased bilateral and monocular presenting VAs of 1.2% and 3.7% in white children 30–71 months of age [22]. It is plausible that the prevailing rate of 92% of referred children due to bilateral causes possibly indicates a developmental trend towards amblyopia as a binocular disorder, consistent with the concept that the fellow eye deficits adapt to prevent excessive interocular difference in order to keep the binocular visual system in balance [29].

### 4.3. Testability, Strengths and Limitations

The LEA Symbols^®^ Pediatric Eye Near and Distance Charts inline logMAR chart obtained a high testability of 98.2% in four-year-old children, comparable to the reported rate in four-year-olds using the standardized inline logMAR chart [20]. Males were more likely to be NT and PT (*p* < 0.001) (Table 3), consistent with the data previously published [9].

Our PT rate of 1.8% paralleled the VIP study results that found Lea Symbols highly testable, with a proportion of 99.5% testable children aged three to five years [11]. However, the VIP study used a modification of the MassVAT form of the Lea Symbols, and not the optotypes inline presented in the gold standard inline logMAR. In the ATS HOTV protocol, testability reached 95% for the 48–54 months age group [10]. It is proposed that HOTV optotypes arranged linearly may be used for children > 5 years [10]. Here, we provide evidence in favor of gold standard inline logMAR charts to be used in everyday clinical practice and for research in four-year-old children.

We consider the large analyzed sample counting 58,712 healthy children to be one of the strengths of the study. However, the sample of children included in comprehensive eye examination and prevalence calculation was rather small and could be considered as a limitation of the study. Thus, the validated result on amblyopia prevalence could not be reached.

To mitigate the potential bias inherent in the discretionary nature of examiner referrals, the patients referred to comprehensive eye examination regardless of their VA pass, based on suspected strabismus and ocular disease at the examiner’s discretion, were excluded from further analysis. This discretion allows the examiner to make subjective judgments based on visual inspection during screening without standardized diagnostic criteria. Different examiners may interpret visual cues differently, leading to inconsistencies in the identification of suspected strabismus or ocular disease. This variability in examiner expertise could result in differential rates of referral, skewing the composition of the study cohort.

In addition to the aforementioned considerations, it is essential to acknowledge the inherent limitations stemming from the sequential assessment of visual acuity, which initiates with near vision and subsequently evaluates distance vision. This approach raises concerns regarding the heightened rates of over-referrals due to factors such as fatigue and limited attention span. While the implementation of randomization could potentially mitigate this phenomenon, it would conflict with the standardization of the CRO-PPEAD, thus presenting a significant challenge. Furthermore, an additional limitation arises from the absence of further investigation with a comprehensive eye examination for the children who successfully pass the initial screening. This represents a missed opportunity to ascertain a more detailed understanding of ocular health status and potentially identify subtle abnormalities that may not be evident during routine screening.

Moreover, while the ZAPS protocol demonstrates efficacy in general, its applicability may be limited for children with cognitive or developmental disabilities, such as those with attention-deficit/hyperactivity disorders, autism, or cerebral palsy. For these individuals, cooperation during screening procedures may be compromised, thereby warranting a referral to a comprehensive ophthalmological examination for a thorough evaluation. Despite these children being encompassed within multidisciplinary team efforts and habilitation processes that incorporate ophthalmic care as an essential component, it is imperative that screening tests for this demographic also adhere to standardized protocols.

## 5. Conclusions

In conclusion, we created uniform standards for screening examination restricted to a four-year-old age group based on the analysis of the large uniform cohort of 58,712 healthy children. Further, the study confirmed the threshold of ≤0.1 logMAR as the cut-off value for unremarkable VA, thereby verifying the normative VA in four-year-old children. Moreover, the study confirmed that when analyzing VA in children ≥ four years of age, inline logMAR optotypes should be used.

The study results enrich our knowledge of normally sighted four-year-old children in terms of VA, but also offer novel insight into the theories involved in normal visual processing and amblyopia development. Further randomized controlled trials and clinical studies on preschool children should embed new protocols to acknowledge the importance of inline spacing, flankers, and stereoacuity [19], as preschool children can still benefit from the plasticity of the critical period of visual brain development.

## Figures and Tables

**Table 1:** Mean visual acuity and area under the curve for all visual acuity tests.

	Binocular Distance	Binocular Near	Right Eye Distance	Right Eye Near	Left Eye Distance	Left Eye Near
Mean VA ± SD	0.83 ± 0.24	0.83 ± 0.24	0.82 ± 0.23	0.82 ± 0.23	0.82 ± 0.24	0.82 ± 0.24
AUC	0.95	0.92	0.97	0.92	0.97	0.92

Abbreviations: VA, visual acuity; SD, standard deviation; AUC, area under the curve.

**Table 2:** The 95% CI and Youden index. Note: The methodology employed for determining the Youden index for each of the parameters is presented in Tables S1–S6.

	Binocular Distance	Binocular Near	Right Eye Distance	Right Eye Near	Left Eye Distance	Left Eye Near
Area	0.95	0.919	0.925	0.923	0.971	0.97
Std error	0.001	0.002	0.002	0.002	0.001	0.001
Asymptotic sig	0	0	0	0	0	0
95% CI low	0.947	0.915	0.922	0.92	0.969	0.968
95% CI upp	0.953	0.923	0.929	0.927	0.973	0.972
Youden index	0.831	0.744	0.784	0.78	0.91	0.91

Abbreviations: std error, standard error; asymptotic sig, asymptotic significance (2-sided); 95% CI, 95% confidence interval.

**Table 3:** Total numbers and percentage of non-testable (NT) and partially testable (PT) four-year-old children. Males were significantly more NT and PT (*p* < 0.001).

	N	Females	Males	*p* Value
Non–testable	2322 (3.05%)	1056 (45.48%)	1266 (54.52%)	*p* < 0.001
Partially–testable	1159 (1.52%)	508 (43.83%)	651 (56.17%)	*p* < 0.001

Abbreviations: N, number of children.

**Table 4:** Mean visual acuity of partially testable four-year-old children (N = 1159).

	BE Near (n = 514)	BE Distance (n = 1010)	RE Near (n = 184)	LE Near (n = 143)	RE Distance (n = 606)	LE Distance (n = 528)
Visual acuity	0.66 ± 0.3	0.73 ± 0.26	0.61 ± 0.28	0.55 ± 0.29	0.68 ± 0.24	0.68 ± 0.24

Abbreviations: N, number of children; n, number of visual acuity tests; BE, both eyes; RE, right eye; LE, left eye.

## Data Availability

The data are available upon request from the corresponding author. The data are not publicly available due to privacy protection.

## Supplementary Materials

The following supporting information can be downloaded at: https://www.mdpi.com/article/10.3390/vision8020039/s1, Figure S1. Croatian Registry of Early Amblyopia Detection screening page.; Figure S2. Croatian Registry of Early Amblyopia Detection complete exam datasheet.; Figure S3. Flowchart of probands sampling.; Figure S4. Distribution of charts used for visual acuity testing.; Figure S5. Distribution of the results of visual acuity testing.; Figure S6. Percentage of children with visual acuity ≤ 0.3. Table S1. Youden index for binocular near. Table S2. Youden index for binocular distance. Table S3. Youden index for right near. Table S4. Youden index for right distance. Table S5. Youden index for left near. Table S6. Youden index for left distance.
