# Supplementary material for: Unveiling Visual Acuity in 58,712 Four-Year-Olds: Standardized Assessment Defined Normative Visual Acuity Threshold
Source: Vision (Basel). 2024 Jun 19;8(2):39. doi: 10.3390/vision8020039 (PMC11209505; doi:10.3390/vision8020039)
Supplement: Supplementary file 1 [file vision-08-00039-s001.zip › vision-3010604-supplementary.pdf]

Unveiling Visual Acuity in 58,712 Four-Year-Olds: Standardized Assessment Defined Normative Visual Acuity Threshold

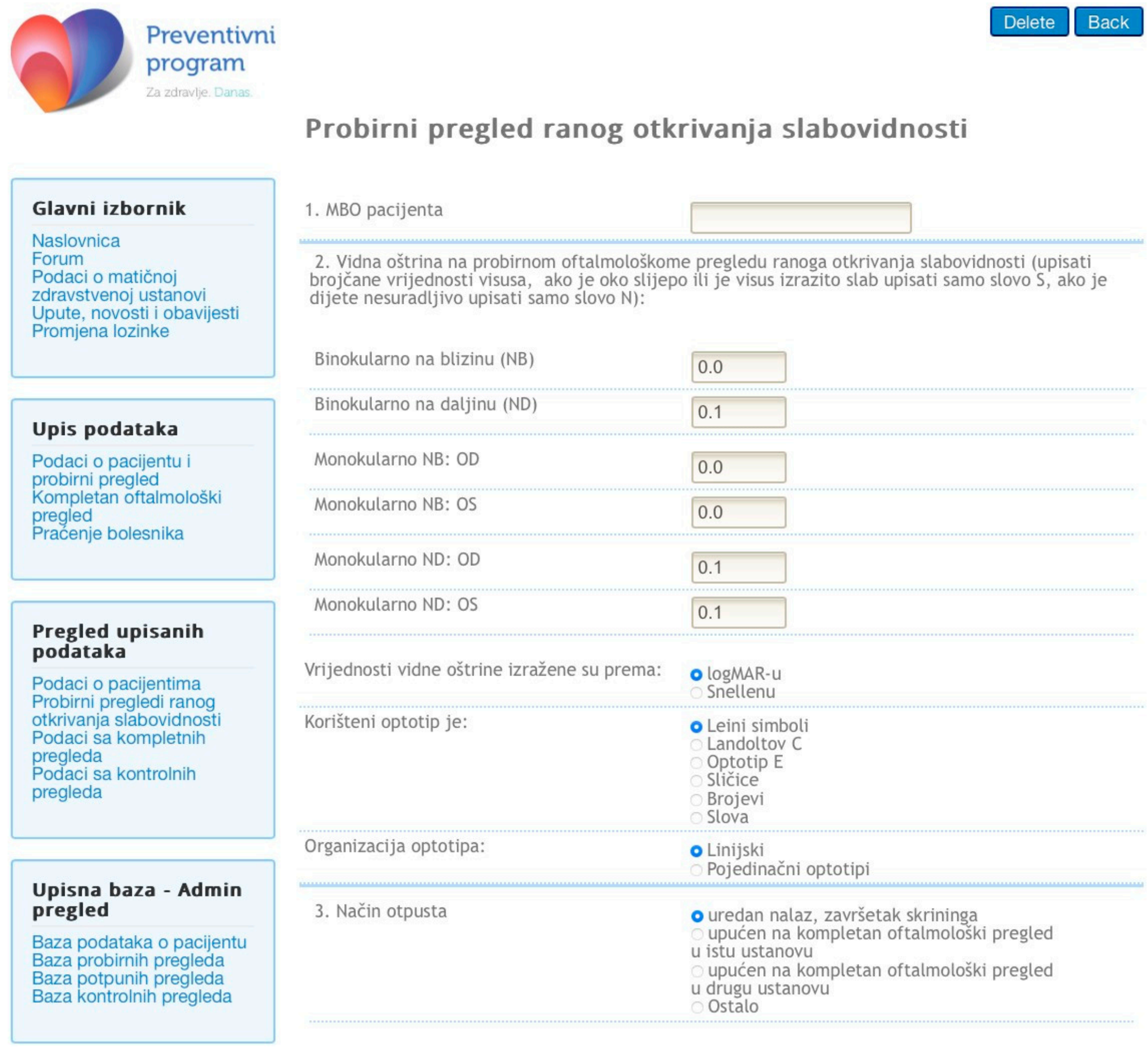

Figure S1. Croatian Registry of Early Amblyopia Detection screening page.

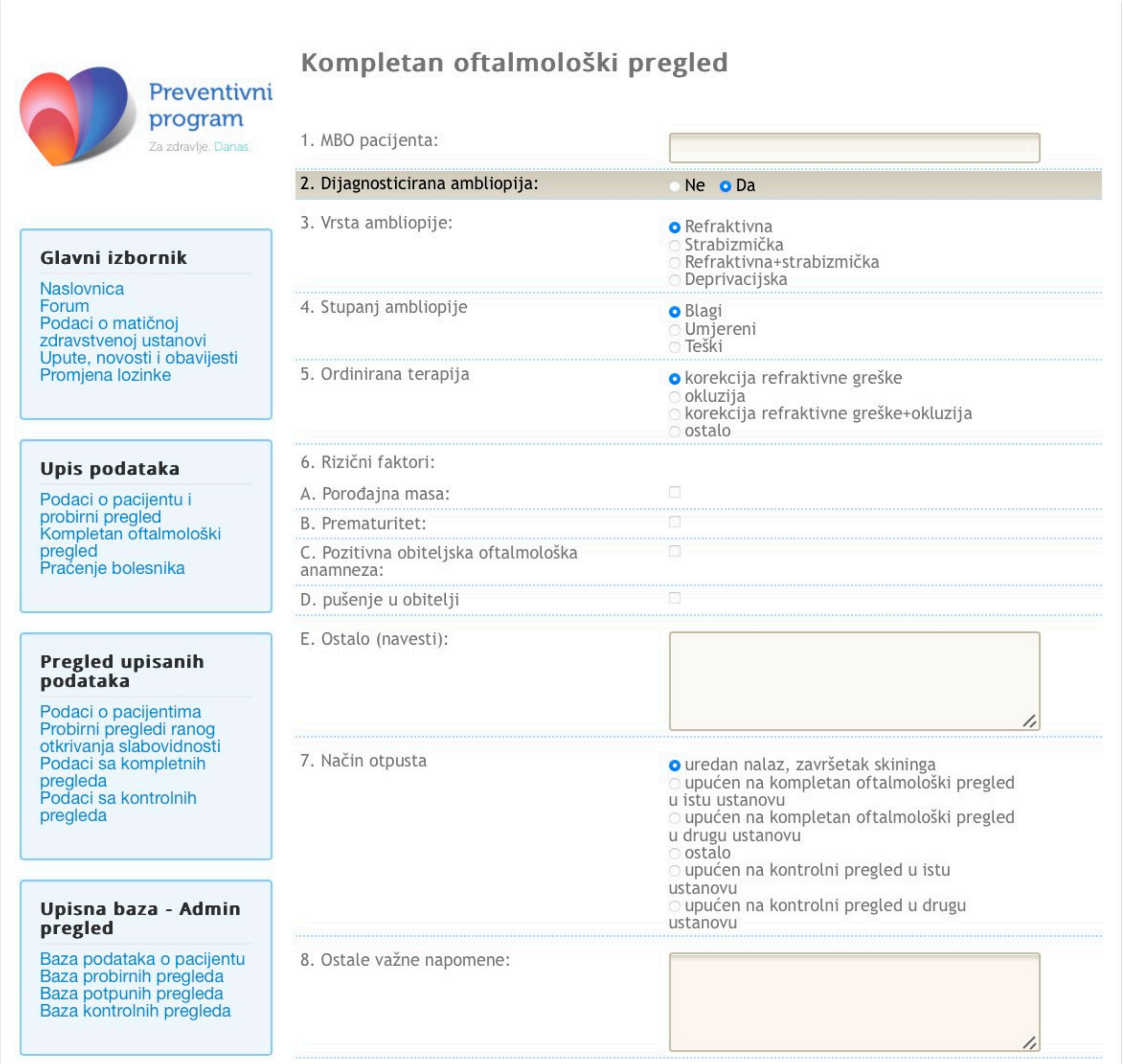

Preventivni program
Za zdravlje. Danas.

Glavni izbornik

Naslovnica
Forum
Podaci o matičnoj zdravstvenoj ustanovi
Upute, novosti i obavijesti
Promjena lozinke

Upis podataka

Podaci o pacijentu i probirni pregled
Kompletan oftalmološki pregled
Praćenje bolesnika

Pregled upisanih podataka

Podaci o pacijentima
Probirni pregledi ranog otkrivanja slabovidnosti
Podaci sa kompletnih pregleda
Podaci sa kontrolnih pregleda

Upisna baza - Admin pregled

Baza podataka o pacijentu
Baza probirnih pregleda
Baza potpunih pregleda
Baza kontrolnih pregleda

Kompletan oftalmološki pregled

1. MBO pacijenta:
2. Dijagnosticirana ambliopija: Ne / Da
3. Vrsta ambliopije: Refraktivna / Strabizmička / Refraktivna+strabizmička / Deprivacijska
4. Stupanj ambliopije: Blagi / Umjereni / Teški
5. Ordinirana terapija: korekcija refraktivne greške / okluzija / korekcija refraktivne greške+okluzija / ostalo
6. Rizični faktori:
A. Porođajna masa:
B. Prematuritet:
C. Pozitivna obiteljska oftalmološka anamneza:
D. pušenje u obitelji
E. Ostalo (navesti):
7. Način otpusta: uredan nalaz, završetak skininga / upućen na kompletan oftalmološki pregled u istu ustanovu / upućen na kompletan oftalmološki pregled u drugu ustanovu / ostalo / upućen na kontrolni pregled u istu ustanovu / upućen na kontrolni pregled u drugu ustanovu
8. Ostale važne napomene:

Figure S2. Croatian Registry of Early Amblyopia Detection complete exam datasheet.

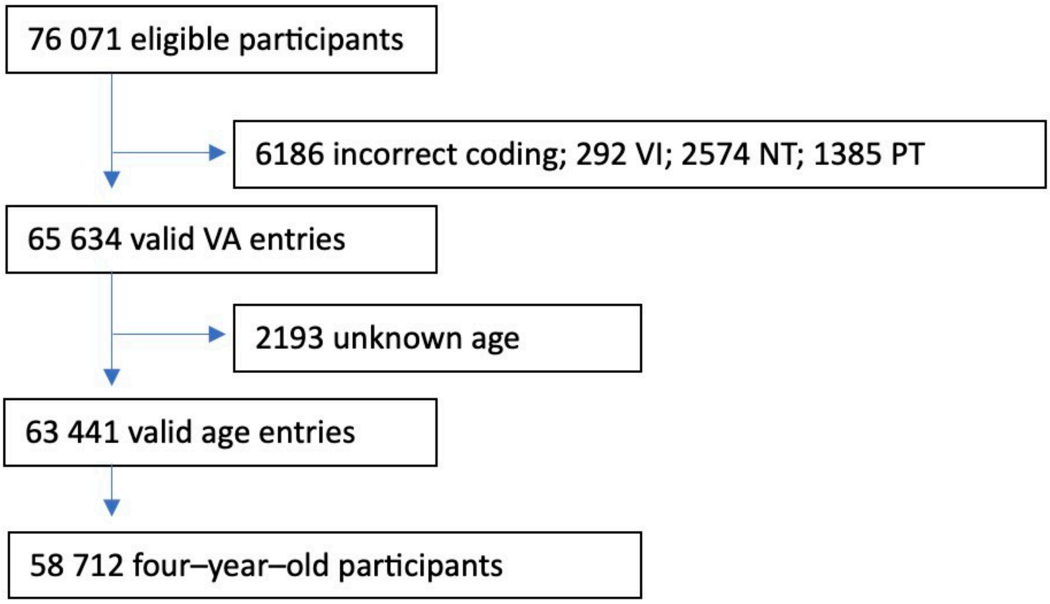

Figure S3. Flowchart of probands sampling. VA, visual acuity; VI, visual impairment; NT, non–testable; PT, partially–testable.

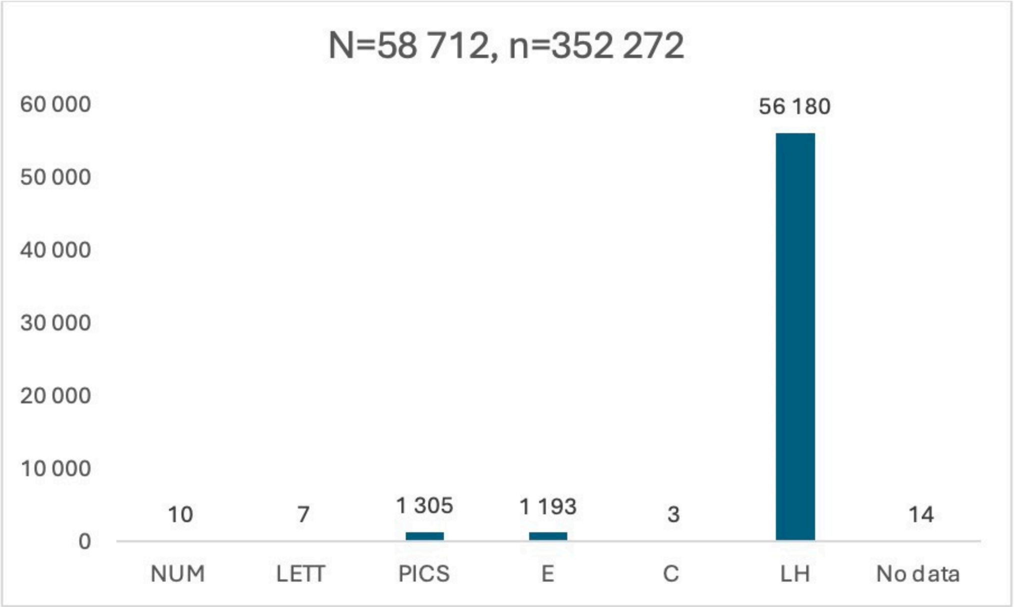

Figure S4. Distribution of charts used for visual acuity testing. N, number of children; n, number of visual acuity exams; LH, Lea inline; C, Landolt's C; E, Tumbling E; NUM, numbers; LETT, letters; PICS, pictures.

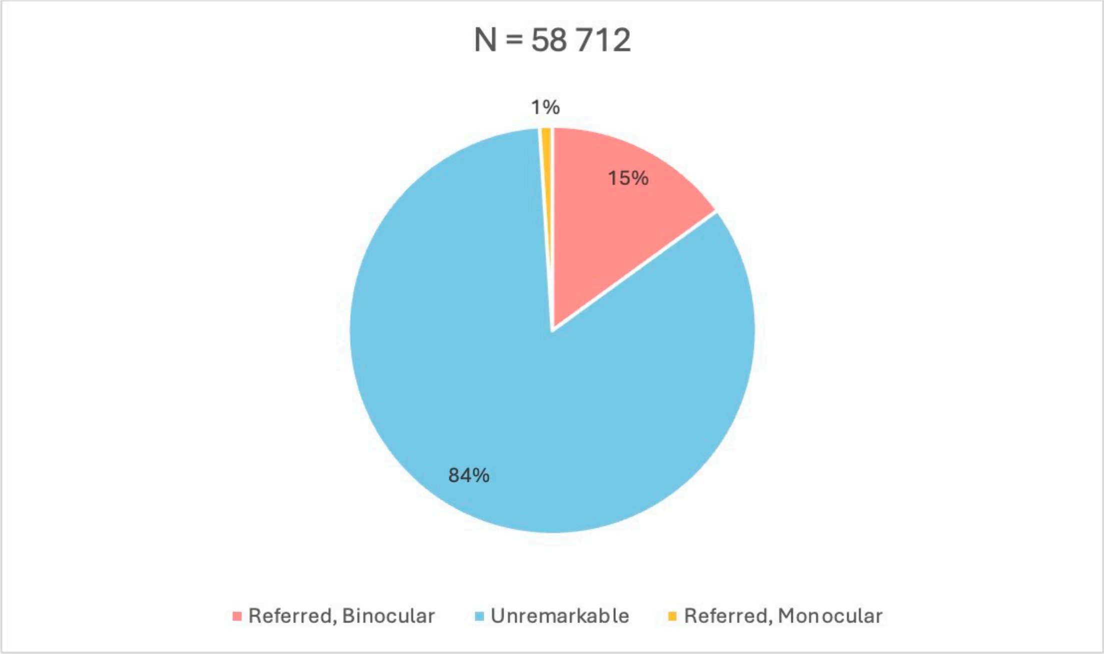

Figure S5. Distribution of the results of visual acuity testing.

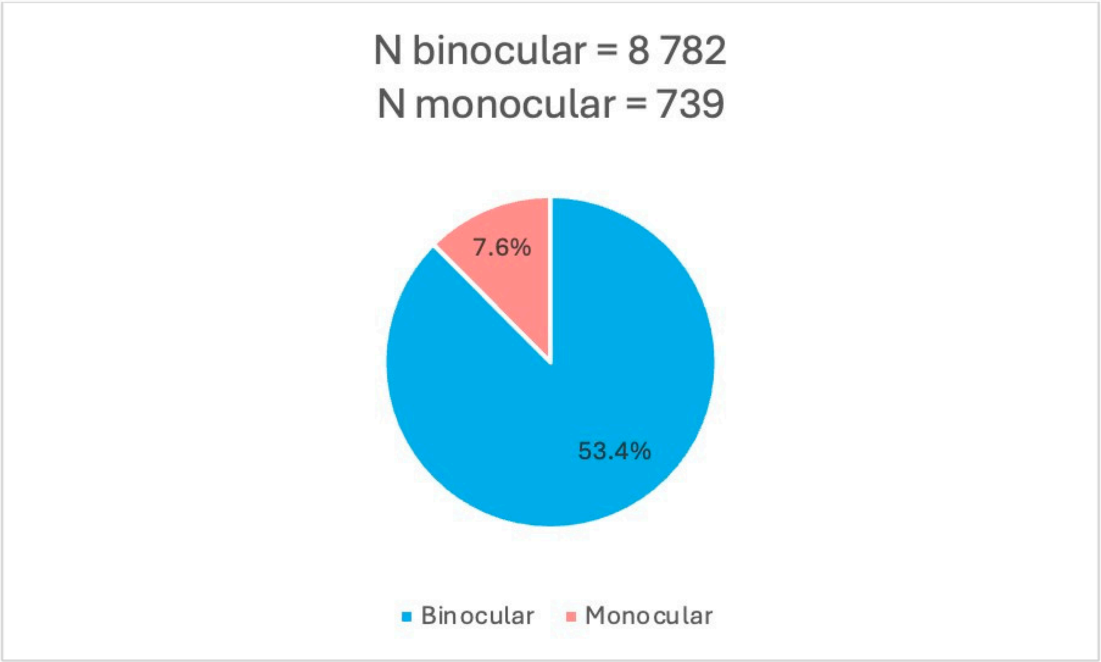

Figure S6. Percentage of children with visual acuity 0.3. N, number of children.

Table S1. Youden index for binocular near.

Positive if Greater Than or Equal To	Sensitivity	1 - Specificity	Youden index
Binocular near			
0.73	0.978	0.234	0.744
0.78	0.978	0.234	0.744
0.66	0.981	0.254	0.727
0.695	0.981	0.254	0.727
0.615	1	0.324	0.676
0.58	1	0.343	0.657
0.555	1	0.344	0.656
0.525	1	0.345	0.655
0.49	1	0.382	0.618
0.425	1	0.386	0.614
0.465	1	0.386	0.614
0.815	0.635	0.068	0.567
0.855	0.635	0.068	0.567
0.885	0.635	0.068	0.567
0.895	0.635	0.068	0.567
0.385	1	0.434	0.566
0.335	1	0.435	0.565
0.36	1	0.435	0.565
0.31	1	0.474	0.526
0.275	1	0.477	0.523
0.925	0.561	0.064	0.497
0.97	0.561	0.064	0.497
0.995	0.561	0.064	0.497
0.24	1	0.503	0.497
0.225	1	0.582	0.418
0.21	1	0.585	0.415
0.18	1	0.606	0.394
0.135	1	0.796	0.204
0.145	1	0.796	0.204
0.155	1	0.796	0.204
0.115	1	0.848	0.152

Table S2. Youden index for binocular distance.

Positive if Greater Than or Equal To	Sensitivity	1 - Specificity	Youden index
Binocular distance			
0.72	0.991	0.16	0.831
0.75	0.991	0.16	0.831
0.78	0.991	0.16	0.831
0.66	0.992	0.192	0.8
0.695	0.992	0.192	0.8
0.61	1	0.287	0.713
0.625	1	0.287	0.713
0.58	1	0.305	0.695
0.525	1	0.307	0.693
0.555	1	0.307	0.693
0.49	1	0.373	0.627
0.42	1	0.378	0.622
0.46	1	0.378	0.622
0.815	0.654	0.046	0.608
0.84	0.652	0.046	0.606
0.875	0.652	0.046	0.606
0.395	1	0.42	0.58
0.37	1	0.422	0.578
0.335	1	0.423	0.577
0.925	0.568	0.034	0.534
0.975	0.568	0.034	0.534
0.31	1	0.474	0.526
0.26	1	0.49	0.51
0.275	1	0.49	0.51
0.29	1	0.49	0.51
0.24	1	0.532	0.468
0.225	1	0.607	0.393
0.21	1	0.631	0.369
0.17	1	0.678	0.322
0.19	1	0.678	0.322
0.155	1	0.873	0.127
0.145	1	0.876	0.124
0.135	1	0.877	0.123
0.105	1	0.915	0.085
0.115	1	0.915	0.085
0.125	1	0.915	0.085
0.075	1	1	0
1.13	0	0	0

Table S3. Youden index for right near.

Positive if Greater Than or Equal To	Sensitivity	1 - Specificity	Youden index
Right near			
0.735	0.966	0.186	0.78
0.78	0.966	0.186	0.78
0.705	0.966	0.187	0.779
0.66	0.971	0.203	0.768
0.695	0.971	0.203	0.768
0.615	1	0.287	0.713
0.58	1	0.312	0.688
0.555	1	0.315	0.685
0.525	1	0.316	0.684
0.49	1	0.374	0.626
0.425	1	0.38	0.62
0.455	1	0.38	0.62
0.47	1	0.38	0.62
0.375	1	0.447	0.553
0.335	1	0.448	0.552
0.31	1	0.505	0.495
0.265	1	0.51	0.49
0.29	1	0.51	0.49
0.895	0.544	0.059	0.485
0.815	0.544	0.06	0.484
0.85	0.544	0.06	0.484
0.88	0.544	0.06	0.484
0.24	1	0.552	0.448
0.925	0.496	0.056	0.44
0.975	0.496	0.056	0.44
0.225	1	0.648	0.352
0.21	1	0.651	0.349
0.18	1	0.675	0.325
0.135	1	0.85	0.15
0.15	1	0.85	0.15
0.105	1	0.878	0.122
0.12	1	0.878	0.122
0.075	1	1	0

Table S4. Youden index for right distance.

Positive if Greater Than or Equal To	Sensitivity	1 - Specificity	Youden index
Right distance			
0.72	0.966	0.182	0.784
0.75	0.966	0.182	0.784
0.78	0.966	0.182	0.784
0.695	0.971	0.199	0.772
0.66	0.971	0.2	0.771
0.615	1	0.288	0.712
0.57	1	0.314	0.686
0.59	1	0.314	0.686
0.555	1	0.317	0.683
0.525	1	0.318	0.682
0.49	1	0.376	0.624
0.425	1	0.382	0.618
0.465	1	0.382	0.618
0.335	1	0.447	0.553
0.37	1	0.447	0.553
0.395	1	0.447	0.553
0.31	1	0.505	0.495
0.895	0.551	0.058	0.493
0.805	0.551	0.059	0.492
0.82	0.551	0.059	0.492
0.86	0.551	0.059	0.492
0.265	1	0.511	0.489
0.29	1	0.511	0.489
0.24	1	0.551	0.449
0.925	0.499	0.055	0.444
0.975	0.499	0.055	0.444
0.225	1	0.647	0.353
0.21	1	0.65	0.35
0.18	1	0.677	0.323
0.135	1	0.852	0.148
0.145	1	0.852	0.148
0.155	1	0.852	0.148
0.105	1	0.882	0.118
0.115	1	0.882	0.118
0.125	1	0.882	0.118
0.04	1	1	0
0.07	1	1	0
0.09	1	1	0

Table S5. Youden index for left near.

Positive if Greater Than or Equal To	Sensitivity	1 - Specificity	Youden index
Left near			
0.72	0.988	0.079	0.909
0.75	0.988	0.079	0.909
0.78	0.988	0.079	0.909
0.695	0.989	0.121	0.868
0.66	0.989	0.122	0.867
0.625	1	0.215	0.785
0.61	1	0.216	0.784
0.58	1	0.242	0.758
0.555	1	0.245	0.755
0.525	1	0.246	0.754
0.49	1	0.351	0.649
0.415	1	0.362	0.638
0.44	1	0.362	0.638
0.465	1	0.362	0.638
0.395	1	0.429	0.571
0.335	1	0.433	0.567
0.37	1	0.433	0.567
0.815	0.563	0.024	0.539
0.84	0.561	0.024	0.537
0.87	0.561	0.024	0.537
0.895	0.561	0.024	0.537
0.94	0.503	0.019	0.484
0.975	0.503	0.019	0.484
0.915	0.503	0.02	0.483
0.31	1	0.527	0.473
0.26	1	0.551	0.449
0.275	1	0.551	0.449
0.29	1	0.551	0.449
0.24	1	0.618	0.382
0.225	1	0.712	0.288
0.21	1	0.731	0.269
0.19	1	0.79	0.21
0.17	1	0.791	0.209
0.155	1	0.921	0.079
0.145	1	0.923	0.077
0.135	1	0.924	0.076
0.11	1	0.953	0.047
0.125	1	0.953	0.047
0.075	1	1	0

Table S6. Youden index for left distance.

Positive if Greater Than or Equal To	Sensitivity	1 - Specificity	Youden index
Left distance			
0.71	0.987	0.077	0.91
0.73	0.987	0.077	0.91
0.75	0.987	0.077	0.91
0.78	0.987	0.077	0.91
0.695	0.989	0.115	0.874
0.66	0.989	0.117	0.872
0.625	1	0.215	0.785
0.61	1	0.216	0.784
0.58	1	0.246	0.754
0.555	1	0.249	0.751
0.525	1	0.25	0.75
0.49	1	0.357	0.643
0.465	1	0.366	0.634
0.42	1	0.367	0.633
0.445	1	0.367	0.633
0.395	1	0.434	0.566
0.335	1	0.439	0.561
0.37	1	0.439	0.561
0.815	0.57	0.024	0.546
0.88	0.568	0.023	0.545
0.895	0.568	0.023	0.545
0.84	0.568	0.024	0.544
0.86	0.568	0.024	0.544
0.925	0.506	0.019	0.487
0.975	0.506	0.019	0.487
0.31	1	0.528	0.472
0.29	1	0.551	0.449
0.26	1	0.552	0.448
0.275	1	0.552	0.448
0.235	1	0.62	0.38
0.245	1	0.62	0.38
0.225	1	0.718	0.282
0.21	1	0.735	0.265
0.19	1	0.794	0.206
0.17	1	0.795	0.205
0.155	1	0.923	0.077
0.145	1	0.924	0.076
0.135	1	0.925	0.075
0.11	1	0.953	0.047
0.125	1	0.953	0.047
